# The Cheltenham Reveille

**Published:** 1891-12

**Authors:** 


THE CHELTENHAM REVEILLE, Vol. III, No. 1, October 1891, Ogontz, Pa. Subscription price, $1.25 per year.

This elegant periodical is published by the boys of the Cheltenham Academy, a well-known school for boys, at Ogontz, Montgomery Co., Penna., about ten miles north of Philadelphia. The present number contains all sorts of miscellaneous information relating to school matters, and is graced with an excellent photographic portrait of the first principal, Rev. Dr. Samuel Clements.

